# Laboratory tools from the Fleming Fund antimicrobial resistance surveillance programme

**DOI:** 10.2471/BLT.25.294565

**Published:** 2026-07-01

**Authors:** Cyril Buhler, Ben Amos, N Claire Gordon, Joanna McKenzie, Sriram Raghu, Natalie Moyen, Emmanuel Azore, Stanley Fenwick, Manisha Bista, Winfred Amia, Areeya Disratthakit, Sheilla Onasanya, Deborah Nanjebe, Heidi Hopkins, Mathias Besong, Antoinette B Ngandijo, Manish Pathak, Toby Leslie

**Affiliations:** a ORDiagnostics, La Genevraye, France.; b Falmouth, England.; c UK Health Security Agency, Porton, England.; d Massey University, Palmerston North, New Zealand.; e New Delhi, India.; f World Organisation for Animal Health, Paris, France.; g Mott MacDonald, 10 Fleet Place, London, EC4M 7RB, England.; h Mott MacDonald, New Delhi, India.; i Mott MacDonald, Kampala, Uganda.; j Mahidol Oxford Tropical Medicine Research Unit, Bangkok, Thailand.; k Mott MacDonald, Accra, Ghana.; l Centre of Expertise in Regulatory Compliance, Quality, Auditing and Safety, Yaoundé, Cameroon.

## Abstract

**Problem:**

Current laboratory assessment tools aimed at improving bacteriology and testing for antimicrobial resistance focus on quality of laboratory services. No guidance exists for establishing bacteriology services necessary for diagnosis and surveillance of antimicrobial resistance in low- and middle-income countries.

**Approach:**

Comprehensive laboratory needs assessment tools and procurement catalogues were developed, piloted and used by the Fleming Fund programme. These tools were designed to support the strengthening of bacteriology testing capacity within national antimicrobial resistance surveillance systems receiving Fleming Fund support.

**Setting:**

The tools were used in reference laboratories and surveillance sites for antimicrobial resistance in 24 countries in the African, South-East Asia and Western Pacific regions, comprising 341 clinical, veterinary, aquaculture, food safety and environmental laboratories.

**Relevant changes:**

The needs assessment tool provides a structured framework for laboratory renovation and installation of bacteriology testing equipment. The procurement catalogues list reagents and consumables for antimicrobial resistance surveillance of priority pathogens in humans in the World Health Organization Global Antimicrobial Resistance and Use Surveillance System and selected bacteria in terrestrial and aquatic food-producing animals. Combined with training and capacity-building initiatives supported through other Fleming Fund investments, these efforts have substantially enhanced diagnostic capacity for bacterial infections and antimicrobial resistance surveillance in the laboratories.

**Lessons learnt:**

These tools were instrumental in identifying gaps to enhance bacteriology laboratory capacity and capabilities and became an essential resource for procurement, tracking supplies and asset management. The tools are adaptable and can strengthen laboratory capacity in settings and health programmes beyond bacteriology and antimicrobial resistance surveillance.

## Résumé

**Problème:**

Les outils d’évaluation de laboratoire actuellement utilisés pour améliorer la bactériologie et le dépistage de la résistance aux antimicrobiens se concentrent sur la qualité des services de laboratoire. Il n’existe aucune recommandation concernant la mise en place de services de bactériologie nécessaires au diagnostic et à la surveillance de la résistance aux antimicrobiens dans les pays à revenu faible ou intermédiaire.

**Approche:**

Des outils complets d’évaluation des besoins des laboratoires ainsi que des catalogues d’approvisionnement ont été élaborés, testés et utilisés dans le cadre du programme du Fonds Fleming. Ils ont été conçus pour soutenir le renforcement des capacités d’analyses bactériologiques au sein de systèmes nationaux de surveillance de la résistance aux antimicrobiens bénéficiant du soutien du Fonds Fleming.

**Environnement local:**

Les outils ont été utilisés dans des laboratoires de référence et des sites de surveillance de la résistance aux antimicrobiens dans 24 pays des régions d’Afrique, d’Asie du Sud-Est et du Pacifique occidental, regroupant 341 laboratoires cliniques, vétérinaires, environnementaux, d’aquaculture et de sécurité alimentaire.

**Changements significatifs:**

L’outil d’évaluation des besoins fournit un cadre structuré pour la rénovation des laboratoires et l’installation d’équipements de tests bactériologiques. Les catalogues d’approvisionnement répertorient les réactifs et les consommables destinés à la surveillance de la résistance aux antimicrobiens des agents pathogènes prioritaires chez l’être humain, dans le cadre du Système mondial de surveillance de la résistance aux antimicrobiens et de leur utilisation de l’Organisation mondiale de la Santé, ainsi que de certaines bactéries présentes chez les animaux terrestres et aquatiques destinés à l’alimentation. Associés aux initiatives de formation et de renforcement des capacités soutenues par d’autres investissements du Fonds Fleming, ces efforts ont considérablement amélioré les capacités de diagnostic des infections bactériennes et de surveillance de la résistance aux antimicrobiens au sein des laboratoires.

**Leçons tirées:**

Ces outils ont joué un rôle déterminant dans l’identification des lacunes à combler pour renforcer les capacités et les compétences des laboratoires bactériologiques et sont devenus une ressource essentielle pour les approvisionnements, le suivi des fournitures et la gestion des ressources. Ils sont adaptables et à même de renforcer les capacités des laboratoires dans des contextes et des programmes de santé qui dépassent le champ de la bactériologie et de la surveillance de la résistance aux antimicrobiens.

## Resumen

**Situación:**

Las herramientas actuales de evaluación de laboratorios destinadas a mejorar la bacteriología y las pruebas de resistencia a los antimicrobianos se centran en la calidad de los servicios de laboratorio. No existen orientaciones para establecer los servicios de bacteriología necesarios para el diagnóstico y la vigilancia de la resistencia a los antimicrobianos en países de ingresos bajos y medios.

**Enfoque:**

En el marco del programa del Fondo Fleming se desarrollaron, pilotaron y utilizaron herramientas integrales para la evaluación de las necesidades de los laboratorios y catálogos de adquisición. Estas herramientas se diseñaron para apoyar el fortalecimiento de la capacidad de realizar pruebas de bacteriología en los sistemas nacionales de vigilancia de la resistencia a los antimicrobianos que reciben apoyo del Fondo Fleming.

**Marco regional:**

Las herramientas se utilizaron en laboratorios de referencia y centros de vigilancia de la resistencia a los antimicrobianos de 24 países de las regiones de África, Asia Sudoriental y el Pacífico Occidental, que comprendían 341 laboratorios clínicos, veterinarios, de acuicultura, de inocuidad alimentaria y ambientales.

**Cambios importantes:**

La herramienta de evaluación de necesidades proporciona un marco estructurado para la renovación de laboratorios y la instalación de equipos para pruebas de bacteriología. Los catálogos de adquisición incluyen reactivos y materiales fungibles para la vigilancia de la resistencia a los antimicrobianos de patógenos prioritarios en seres humanos dentro del Sistema Mundial de Vigilancia de la Resistencia a los Antimicrobianos y de su Uso de la Organización Mundial de la Salud, así como de bacterias seleccionadas en animales terrestres y acuáticos destinados a la producción de alimentos. Combinadas con las iniciativas de formación y fortalecimiento de capacidades financiadas mediante otras inversiones del Fondo Fleming, estas acciones han reforzado considerablemente la capacidad diagnóstica de las infecciones bacterianas y la vigilancia de la resistencia a los antimicrobianos en los laboratorios.

**Lecciones aprendidas:**

Estas herramientas fueron fundamentales para identificar deficiencias y fortalecer la capacidad y las competencias de los laboratorios de bacteriología, y se convirtieron en un recurso esencial para la adquisición, el seguimiento de suministros y la gestión de activos. Estas herramientas son adaptables y pueden fortalecer la capacidad de los laboratorios en otros contextos y programas de salud, más allá de la bacteriología y de la vigilancia de la resistencia a los antimicrobianos.

## ملخص

**المشكلة:**

إن أدوات تقييم المختبرات الحالية، والتي تهدف إلى تحسين علم البكتيريا واختبار مقاومة مضادات الميكروبات، إنما تركز على جودة خدمات المختبرات. ليست هناك إرشادات لإنشاء خدمات علم البكتيريا اللازمة لتشخيص ومراقبة مقاومة مضادات الميكروبات في الدول ذات الدخل المنخفض والدخل المتوسط.

**الأسلوب:**

تم تطوير أدوات شاملة لتقييم احتياجات المختبرات وكتالوجات التوريدات، وتجريبها واستخدامها بواسطة برنامج صندوق فليمنج. تم تصميم هذه الأدوات لدعم تعزيز قدرات اختبار علم البكتيريا، ضمن أنظمة مراقبة مقاومة مضادات الميكروبات الوطنية، التي تتلقى دعمًا من صندوق فليمنج.

**المواقع المحلية:**

استُخدمت هذه الأدوات في المختبرات المرجعية ومواقع مراقبة مقاومة مضادات الميكروبات في 24 دولة في أفريقيا وجنوب شرق آسيا وغرب المحيط الهادئ، وتضم 341 مختبرًا إكلينيكيًا وبيطريًا ومائيًا، ومختبرًا لسلامة الأغذية، ومختبرًا بيئيًا.

**التغيّرات ذات الصلة:**

توفر أداة تقييم الاحتياجات إطارًا عمل منظم لتجديد المختبرات وتركيب معدات اختبار علم البكتيريا. تُدرج كتالوجات التوريدات الكواشف والمواد الاستهلاكية اللازمة لرصد مقاومة المضادات الحيوية لدى مسببات الأمراض ذات الأولوية لدى البشر، وذلك ضمن النظام العالمي لرصد مقاومة المضادات الحيوية واستخدامها التابع لمنظمة الصحة العالمية، بالإضافة إلى أنواع مختارة من البكتيريا الموجودة في الحيوانات البرية والمائية المنتجة للغذاء. وبالتزامن مع مبادرات التدريب وبناء القدرات المدعومة من خلال استثمارات أخرى لصندوق فليمنج، ساهمت هذه الجهود بشكل كبير في تعزيز القدرات التشخيصية للعدوى البكتيرية ورصد مقاومة المضادات الحيوية في المختبرات.

**الدروس المستفادة:**

كانت هذه الأدوات أساسية في تحديد الثغرات لتعزيز قدرات وإمكانيات مختبرات علم البكتيريا، وأصبحت مورداً بالغ الأهمية للتوريدات وتتبع الإمدادات وإدارة الأصول. تتميز هذه الأدوات بقابليتها للتكيف، ويمكنها تعزيز القدرات المختبرية في مختلف البيئات والبرامج الصحية، بما يتجاوز علم البكتيريا ورصد مقاومة المضادات الحيوية.

## 摘要

**问题:**

目前的旨在完善细菌学和抗微生物药物耐药性检测的实验室评估工具非常注重实验室服务的质量。目前没有相关指导文件可供中低收入国家构建用来诊断和监测抗微生物药物耐药性的细菌学服务。

**方法:**

弗莱明基金研发了实验室综合需求评估工具和采购目录，完成试点并投入使用。这些工具旨在支持接受弗莱明基金支持的国家级抗微生物药物耐药性监测系统提高细菌学检测能力。

**当地状况:**

非洲、东南亚和西太平洋地区的 24 个国家用于抗微生物药物耐药性的参比实验室和监测站点都在使用这些工具，覆盖 341 个临床、兽医、水产、食品安全及环境实验室。

**相关变化:**

需求评估工具为实验室改造和安装细菌学检测设备提供了结构框架。采购目录列出了世卫组织全球抗微生物耐药性与抗菌药物使用监测系统进行人体重点病原体耐药监测，以及陆生与水生食用动物特定致病菌耐药监测所需的各类试剂与耗材。结合通过其他弗莱明基金投资资源支持的培训和能力建设举措，这些工作大幅提高了实验室的细菌感染诊断能力和抗微生物药物耐药性的监测水平。

**经验教训:**

这些工具对找出提升细菌学实验室能力与水平方面的不足方面起关键作用，成为采购、耗材跟踪和资产管理的必备资源。这些工具可灵活适配，可以加强实验室在除了细菌学和抗微生物药物耐药性监测以外的其他工作场景和卫生项目中的能力。

## Резюме

**Проблема:**

Современные инструменты лабораторной оценки, направленные на повышение эффективности бактериологических исследований и тестирования на устойчивость к противомикробным препаратам, ориентированы на качество лабораторных услуг. В странах с низким и средним уровнем дохода отсутствуют руководства по организации бактериологических служб, необходимых для диагностики и эпиднадзора за устойчивостью к противомикробным препаратам.

**Подход:**

В рамках программы Фонда Флеминга были разработаны, опробованы и внедрены инструменты комплексной оценки лабораторных потребностей и каталоги закупок. Эти инструменты предназначены для укрепления потенциала бактериологического тестирования в национальных системах эпиднадзора за устойчивостью к противомикробным препаратам, получающих поддержку от Фонда Флеминга.

**Местные условия:**

Эти инструменты использовались в референс-лабораториях и пунктах эпиднадзора за устойчивостью к противомикробным препаратам в 24 странах Африки, Юго-Восточной Азии и Западной части Тихого океана. В общей сложности была задействована 341 лаборатория. Типы лабораторий включали клинические, ветеринарные, аквакультурные, экологические лаборатории и лаборатории по безопасности пищевых продуктов.

**Осуществленные перемены:**

Инструмент оценки потребностей предоставляет четкую структуру для модернизации лабораторий и установки оборудования для бактериологического тестирования. Каталоги закупок содержат списки реагентов и расходных материалов для эпиднадзора за устойчивостью к противомикробным препаратам у человека в рамках Глобальной системы Всемирной организации здравоохранения по эпиднадзору за устойчивостью к противомикробным препаратам и их применением (GLASS), а также за отдельными бактериями у наземных и водных сельскохозяйственных животных. В сочетании с инициативами по обучению и развитию потенциала, реализуемыми за счет других инвестиций Фонда Флеминга, эти усилия существенно расширили возможности лабораторий в области диагностики бактериальных инфекций и эпиднадзора за устойчивостью к противомикробным препаратам.

**Выводы:**

Данные инструменты помогли выявить пробелы для расширения потенциала и возможностей бактериологических лабораторий и стали важным ресурсом для осуществления закупок, отслеживания поставок и управления активами. Эти инструменты легко адаптируются и могут укрепить потенциал лабораторий в других условиях и программах здравоохранения, выходящих за рамки бактериологии и эпиднадзора за устойчивостью к противомикробным препаратам.

## Introduction

Clinical bacteriology testing capacity and capability in resource-constrained settings are needed for accurate diagnosis and treatment of bacterial infections, effective antimicrobial stewardship activities and data collection for surveillance of antimicrobial resistance.^1^ However, a recent study in sub-Saharan Africa showed important gaps in coverage, volume and quality of bacteriology testing. Only 1.3% (675/53770) of clinical laboratories in 14 countries provided bacteriology testing,^2^ with only 393 facilities undertaking antimicrobial susceptibility testing and 66.9% (263/393) of these facilities performing fewer than 1000 tests for antimicrobial susceptibility a year. The World Health Organization’s (WHO) people-centred approach^3^ emphasizes diagnostic coverage and capabilities for bacterial and fungal pathogens as a priority.

Laboratory assessment tools are often used to support laboratories in resource-constrained settings in improving their capacity and capability. In bacteriology, these tools include the United States Centers for Disease Control and Prevention Laboratory Assessment of Antibiotic Resistance Testing Capacity^4^ and the Foundation for Innovative New Diagnostics antimicrobial resistance scorecards.^5^ These tools are designed for use in established laboratories where basic capability is already in place and the quality of testing needs to be assessed. Little guidance is available for ministries of health, agriculture and environment on establishing basic bacteriology services in low- and middle-income countries where these diagnostic services are lacking.

Since 2017, the Fleming Fund has supported countries to develop systems for antimicrobial resistance surveillance. A large proportion of funds is used for laboratory development and improvement including renovation, refurbishment, equipment and consumables for bacteriological diagnostics. This support is offered across the One Health disciplines of human health, veterinary, food safety and environmental microbiology laboratories.

To ensure consistent support for laboratories, the Fleming Fund Management Agent developed a laboratory needs assessment tool to evaluate the infrastructure and equipment required to provide effective and safe bacteriology services. Concurrently, procurement catalogues for key reagents and consumables were developed focusing on the WHO Global Antimicrobial Resistance and Use Surveillance System (GLASS) priority pathogens list^6^ and indicator pathogens in poultry.^7^ This paper outlines the main components of a laboratory needs assessment tool and the procurement catalogues developed by the Fleming Fund and the lessons learnt during deployment.

## Local settings

The tools were used in antimicrobial resistance reference laboratories and surveillance site laboratories in 24 Fleming Fund-supported countries in the African, South-East Asia and Western Pacific regions. By the end of the programme, 341 clinical, veterinary, aquaculture, food safety and environmental laboratories had used the tool. In the human health sector, sites provided diagnostic services to health facilities, whereas veterinary sites were primarily for conducting active surveillance activities. 

## Approach

The Fleming Fund Management Agent developed a pilot tool in 2017 to assess infrastructure, utilities, equipment and consumable requirements for a basic bacteriology laboratory that can perform bacterial isolation and identification of non-fastidious pathogens from blood and urine samples and antimicrobial susceptibility testing by disc diffusion testing.^8^ The tool was designed to be completed by laboratory assessors from the Fleming Fund Management Agent alongside staff from the recipient laboratory. The tool was piloted in Ghana, Lao People’s Democratic Republic, Nepal and Uganda, in 28 human health and 13 animal health laboratories.

Based on the pilot, the tool was modified to align more closely with the WHO *Global Antimicrobial Resistance Surveillance System: manual for early implementation*^9^ and the London School of Tropical Medicine & Hygiene roadmap for participation in GLASS.^8^ The sections modified were on biosafety and biosecurity, waste management, power and water, fixtures, equipment (including the working status and maintenance), consumables, information technology and data management, quality management, and clinical liaison and reporting. The tool included a mechanism to estimate the requirements for equipment and instruments based on actual or projected throughput of specimens at each site. 

Further versions of the tool were developed during ongoing programme implementation, particularly to address basic structural requirements (e.g. doors, windows and flooring), with the latest tool published in 2025.^10^ Each of the following subsections describes one part of the assessment tool.

### Premises assessment

**Fig. 1:**
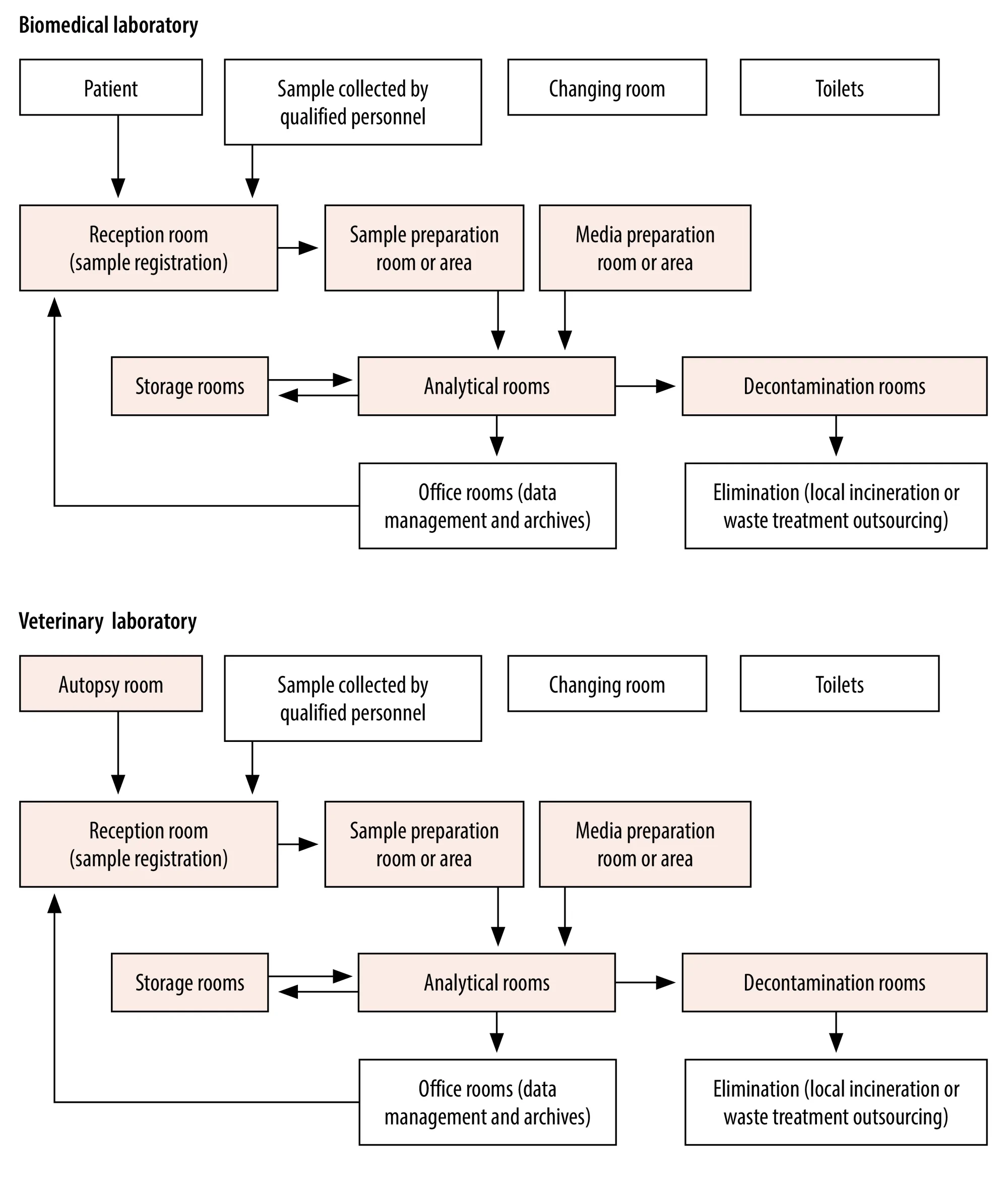
Schematic layouts of rooms and areas evaluated in the laboratory needs assessment tool for biomedical laboratories and veterinary laboratories

Note: coloured boxes indicate areas or rooms under consideration for refurbishment and equipment needs assessment.

The laboratory needs assessment tool provides a structured framework for evaluating the needs of a laboratory facility to ensure a safe and secure testing environment. The assessment covers all aspects of the physical laboratory environment including work areas, benches, walls, ceilings, floors, doors, windows and climate control. Dedicated rooms, such as media preparation and autopsy rooms in veterinary facilities, are also assessed (Fig. 1). Rooms not directly related to laboratory work are excluded from the assessment (e.g. common areas of the laboratories). Major renovations (e.g. construction of new buildings or substantial internal changes to layout) are not included in the tool as this type of major building work is not undertaken by the Fleming Fund.

Trained evaluators assess the adequacy of laboratory space based on current and projected testing volumes and staffing levels. The tool also collects information on the facility’s biosafety and biosecurity infrastructure. The tool is not intended to replace a comprehensive risk assessment as recommended by WHO;^11^ rather, it helps identify the equipment needed to handle routine clinical specimens at biosafety level 2.

### Utilities assessment

The tool includes assessments of essential but often overlooked laboratory utilities, particularly electricity and water systems. The tool assesses the presence of an adequate and stable electricity supply to support general laboratory equipment and analysers, including uninterruptible power supply back-up power in the event of a mains outage.

The supply of high-quality water to meet bacteriology testing standards is also assessed. Acknowledging that infrastructure needs vary by facility, the tool is intentionally adaptable, with a recommendation for a formal electrical survey and further specialist assessment where the minimum standard is not met. This recommendation ensures renovation plans are technically sound, context-specific, and budgeted appropriately, supporting sustainable improvements aligned with each facility’s operational needs and country specification.

### Equipment list

The tool assesses general laboratory apparatus essential for: (i) preparing quality-assured media; (ii) culturing and isolating bacterial pathogens; (iii) performing antimicrobial susceptibility testing; and (iv) storing reagents and culture isolates. Equipment for data management, analysis and communication of surveillance and hospital data is also assessed. The list is similar to previous equipment lists^12^ but emphasizes the importance of maintenance and calibration contract services for general laboratory equipment, including biosafety cabinets, incubators and autoclaves. The minimum standard, in line with the WHO GLASS programme, is a capability for blood culture (preferably using an automated system) and urine culture, with antimicrobial susceptibility testing performed by disc diffusion. Higher throughput, well-resourced laboratories or national laboratories were also assessed for suitability for advanced systems such as automated antimicrobial susceptibility testing platforms and matrix-assisted laser desorption ionization time-of-flight mass spectrometry for organism identification.

### Summary of needs

All responses that identify a specific need are automatically compiled into a summary page, which serves as the basis for developing a costed site plan. The tool also provides technical specifications of the different items and their intended use to streamline the procurement process.

### Procurement catalogues

To further assist in strengthening surveillance of antimicrobial resistance, standardized procurement catalogues of reagents and key consumables were developed for both the human health^13^ and the veterinary sectors.^14^ The catalogues are organized into sections covering general culture media, essential chemical reagents, standard biochemical reagents and kits for bacterial identification, and reagents specific for antimicrobial susceptibility testing. For the veterinary sector, the catalogue includes a section on consumables for sample collection.

Since a standard catalogue cannot cover every testing possibility, the reagents proposed are recommendations based on key guiding principles. First, wherever possible, the tests included are simple, affordable and suitable for resource-constrained settings. Second, most reagents should be accessible from local distributors, as sustainable sourcing is a recurrent problem in low- and middle-income countries.^1^ Third, the United Kingdom Standards for Microbiology Investigations^15^ was the main reference to ensure alignment with recognized guidelines. Fourth, the catalogues cover the list of GLASS priority pathogens for the human health sector^6^ and *Escherichia coli*, *Salmonella*, *Campylobacter* and *Enterococcus* for the veterinary sector.^7^ The catalogues have been kept up to date with newer lists of priority organisms (e.g. from WHO GLASS). These procurement catalogues also allowed for diagnosis of locally important bacterial pathogens not covered by the GLASS pathogen priority list.

## Relevant changes

The laboratory needs assessment tool and procurement catalogues identified important gaps in laboratory capacity in microbiology services at 341 supported sites. The tool provided a list of essential equipment along with the modifications required to install and operate them as part of a robust and resilient laboratory system. The planning tool provided the basis for resource allocation for laboratory strengthening across the Fleming Fund programme, supporting important improvements in surveillance of antimicrobial resistance.

In some countries, assessments using the tool highlighted significant gaps in technical microbiology knowledge. As a result, targeted capacity-building initiatives were arranged by Fleming Fund grantees, with experts providing training in both basic and advanced microbiology diagnostics.

## Lessons learnt

Box 1 provides a summary of the main lessons learnt in using the laboratory needs assessment tool and procurement catalogues. Challenges included language barriers between site personnel and external stakeholders (including engineers, procurement teams and regional programme staff), administrative hurdles in organizing site visits and limited time to cover the wide range of topics in the tool. The detailed requirements of bacteriology assessments meant that a single facility typically required at least a full day and a team of laboratory experts, possibly including a biomedical engineer. Sharing the tools with laboratory managers in advance proved most effective, enabling them to prepare beforehand. The catalogues provided a list of critical reagents and consumables but more accurate quantification would help forecast testing volumes and cost.

Box 1 **Summary of main lessons learnt**

- The laboratory needs assessment tool was able to identify gaps in laboratory capacity and guide equipment acquisition and laboratory renovation to establish foundational microbiology services.
- The procurement catalogues are often the most comprehensive guide for reagents and consumables procurement available to stakeholders involved in bacteriology diagnostic and antimicrobial resistance surveillance in low-resource settings.
- To ensure a thorough assessment, the participation of laboratory experts, domain specialists (e.g. veterinarians or environmental microbiologists) and engineers is needed to evaluate essential infrastructure such as electrical and water systems.

The laboratory needs assessment tool can be adapted for use in other laboratory evaluations beyond those of the Fleming Fund, and is a demonstrably versatile resource for strengthening laboratory and surveillance systems. The procurement catalogues were invaluable resources, serving as the first comprehensive and accessible guide to microbiology reagents and key consumables for many users in resource-constrained settings.

## References

[1] Ombelet S, Ronat JB, Walsh T, Yansouni CP, Cox J, Vlieghe E, et al.; Bacteriology in Low Resource Settings working group. Clinical bacteriology in low-resource settings: today’s solutions. Lancet Infect Dis. 2018 Aug;18(8):e248–58. 10.1016/S1473-3099(18)30093-8 29519767

[2] Ondoa P, Kapoor G, Alimi Y, Shumba E, Osena G, Maina M, et al.; MAAP study group. Bacteriology testing and antimicrobial resistance detection capacity of national tiered laboratory networks in sub-Saharan Africa: an analysis from 14 countries. Lancet Microbe. 2025 Jan;6(1):100976. 10.1016/j.lanmic.2024.100976 39653051

[3] People-centred approach to addressing antimicrobial resistance in human health: WHO core package of interventions to support national action plans. Geneva: World Health Organization; 2023. Available from: https://iris.who.int/handle/10665/373458 [cited 2026 Jun 15].

[4] Laboratory Assessment of Antibiotic Resistance Testing Capacity. Atlanta: US Centers for Disease Control and Prevention; 2020. Available from: https://www.cdc.gov/antimicrobial-resistance/php/toolkit/?CDC_AAref_Val=https://www.cdc.gov/drugresistance/intl-activities/laarc.html [cited 2026 Jun 15].

[5] AMR laboratory scorecards package accessed [internet]. Geneva: FIND; 2021. Available from: https://www.finddx.org/tools-and-resources/policy-support-and-guidance/locally-tailored-guidance-to-enable-implementation/amr-laboratory-scorecard-package/ [cited 2026 Jun 15].

[6] WHO bacterial priority pathogens list, 2024: bacterial pathogens of public health importance to guide research, development and strategies to prevent and control antimicrobial resistance. Geneva: World Health Organization; 2024. Available from https://iris.who.int/handle/10665/376776 [cited 2026 Jun 15].

[7] A protocol for active AMR surveillance in poultry. London: The Fleming Fund; 2019. 10.48060/tghn.191

[8] Seale AC, Gordon NC, Islam J, Peacock SJ, Scott JAG. AMR surveillance in low- and middle-income settings – a roadmap for participation in the Global Antimicrobial Surveillance System (GLASS). Wellcome Open Res. 2017 Sep 26;2:92. 10.12688/wellcomeopenres.12527.1 29062918 PMC5645727

[9] Global antimicrobial resistance surveillance system: manual for early implementation. Geneva: World Health Organization; 2015. Available from: https://iris.who.int/handle/10665/188783 [cited 2026 Jun 15].

[10] Laboratory needs assessment tool (LNAT): infrastructure utilities and equipment. London: The Fleming Fund; 2025. 10.48060/tghn.181

[11] Laboratory biosafety manual. 4th ed. Geneva: World Health Organization; 2020. Available from: https://iris.who.int/handle/10665/337956 [cited 2026 Jun 15].

[12] Roberts T, Luangasanatip N, Ling CL, Hopkins J, Jaksuwan R, Lubell Y, et al. Antimicrobial resistance detection in Southeast Asian hospitals is critically important from both patient and societal perspectives, but what is its cost? PLOS Glob Public Health. 2021 Oct 13;1(10):e0000018. 10.1371/journal.pgph.0000018 34746931 PMC7611947

[13] Procurement catalogue – reagents and consumables for human health facilities. London: The Fleming Fund; 2025. 10.48060/tghn.182

[14] Procurement catalogue – reagents and consumables for veterinary laboratories. London: The Fleming Fund; 2025. 10.48060/tghn.183

[15] UK Standards for Microbiology Investigations [internet]. London: The Royal College of Pathologists; 2026. Available from: https://www.rcpath.org/profession/publications/standards-for-microbiology-investigations.html [cited 2026 Jun 15].

